# Supplementary material for: Computational design of prefusion-stabilized Herpesvirus gB trimers
Source: bioRxiv. 2024 Oct 24:2024.10.23.619923. Preprint. [Version 1] doi: 10.1101/2024.10.23.619923 (PMC11526958; doi:10.1101/2024.10.23.619923)
Supplement: Supplement 1 [file NIHPP2024.10.23.619923v1-supplement-1.pdf]

Table S2. Cryo-EM data collection, refinement and validation statistics for EBV gB constructs.

	gB-deli	gB-Δ	gB-deD	gB-deliDoP-I5350A	EBV gB-deliDoP-I5350A2h				EBV gB-deliDoP-I5350A2hN			
**Data collection and processing**												
Magnification (nominal)	130,000	130,000	130,000	45,000	130,000				130,000			
Voltage (kV)	300	300	300	200	300				300			
Electron exposure ($e^-/Å^2$)	63	63	63	47	63				63			
Defocus range (μm)	0.3-2.0	0.3-2.0	0.3-2.0	0.3-2.0	0.3-2.0				0.3-2.0			
Pixel size (Å)	0.843	0.843	0.843	0.89	0.829				0.829			
Processing Type	Global	Global	Global	Global	Global	Local I5350A2h	Local gB DII-DV	Local gB DIII-DV	Global	Local I5350A2hN	Local gB DI-DV	Local gB DII-DV
Symmetry imposed	*C3*	*C3*	*C1*	*C1*	*C1*	*C3*	*C1*	*C3*	*C1*	*C3*	*C1*	*C3*
Initial particle images (no.)	88,939	135,291	1,129,600	32,268	113,595	64,249	64,249	64,249	287,619	155,157	155,157	155,157
Final particle images (no.)	22,385	56,843	324,509	18,033	64,249	64,249	64,249	64,249	155,157	155,157	155,157	155,157
Map resolution (Å)	3.5	5.7	4.1	9.2	4.4	3.7	4.4	4.0	3.4	2.7	3.1	2.9
FSC threshold	0.143	0.143	0.143	0.143	0.143	0.143	0.143	0.143	0.143	0.143	0.143	0.143
Map sharpening *B* factor ($Å^2$)	-94	-547	-168	-1001	-141	-125	-144	-138	-98	-96	-92	-85
**Model Refinement**												
Initial models used (PDB codes)	3FVC	3FVC	3FVC	-	3FVC; 7KDP				3FVC; 7KDP			
Model resolution (Å)	3.8	8.2*	6.8	-	6.6	3.8	4.7	1.5	3.5	2.8	3.4	3.0
FSC threshold	0.5	0.5	0.5	-	0.5	0.5	0.5	0.5	0.5	0.5	0.5	0.5
Model composition												
Nonhydrogen atoms	12,057	2,643	6,375	-	12,935	5,427	7,094	4,167	16,604	5,592	12,331	8,406
Protein residues	1,590	516	1083	-	2,470	774	1,109	636	2,409	762	1,624	1083
Glycan residues	24	3	13	-	27	0	20	3	39	0	36	24
B factors ($Å^2$)												
Protein	42	160	172	-	212	36	136	63	65	20	38	11
Glycans	48	140	167	-	246	-	140	74	105	-	58	19
R.m.s. deviations												
Bond lengths (Å)	0.01	0.01	0.01	-	0.01	0.01	0.01	0.01	0.01	0.01	0.01	0.01
Bond angles (°)	0.90	0.84	1.01	-	0.98	1.02	1.01	0.92	0.96	0.92	0.95	1.15
**Model Validation**												
MolProbity score	0.6	0.6	0.9	-	0.8	0.8	1.1	1.2	0.9	0.6	1.1	0.7
Clashscore	0.2	0.3	1.8	-	0.9	0.8	2.4	3.8	1.7	0.4	2.9	0.7
Rotamer outliers(%)	0.0	0.0	0.0	-	1.1	1.3	0.3	0.0	0.2	0.0	0.2	0.0
Ramachandran plot												
Favored (%)	99	99	98	-	98	99	98	98	99	99	99	99
Allowed (%)	1	1	2	-	2	1	2	2	1	1	1	1
Outliers (%)	0	0	0	-	0	0	0	0	0	0	0	0
EMRinger score	3.7	N/A*	2.4	-	2.2	3.0	2.9	3.2	3.9	3.9	3.7	4.4
**Data Availability**												
EMDB	XXXX	XXXX	XXXX	XXXX	XXXX	XXXX	XXXX	XXXX	XXXX	XXXX	XXXX	XXXX
PDB	XXXX	XXXX	XXXX	-	XXXX	XXXX	XXXX	XXXX	XXXX	XXXX	XXXX	XXXX

*most side-chains not built in model reducing reliability of EMRinger score and model resolution calculation

bioRxiv preprint doi: https://doi.org/10.1101/2024.10.23.619923; this version posted October 24, 2024. The copyright holder for this preprint (which was not certified by peer review) is the author/funder, who has granted bioRxiv a license to display the preprint in perpetuity. It is made available under aCC-BY 4.0 International license.

Table S3. Cryo-EM data collection, refinement and validation statistics for SARS-CoV-2 S 2P oP3h constructs.

	**SARS-CoV-2 S 2P oP3h (3X RINEIER)**		**SARS-CoV-2 S 2P oP3h (4X RINAIET)**	
**Data collection and processing**				
Magnification (nominal)	45,000		45,000	
Voltage (kV)	200		200	
Electron exposure (e⁻/Å^2)	47		47	
Defocus range (μm)	0.3-2.0		0.3-2.0	
Pixel size (Å)	0.89		0.89	
Processing Type	Global	Local (oP3h)	Global	Local (oP3h)
Symmetry imposed	C3	C3	C3	C3
Initial particle images (no.)	59,434	34,709	120,164	86,822
Final particle images (no.)	34,709	34,709	86,822	86,822
Map resolution (Å)	3.4	3.6	2.8	3.1
FSC threshold	0.143	0.143	0.143	0.143
Map sharpening *B* factor (Å^2)	-85	-110	-85	-144
**Model Refinement**				
Initial model used (PDB code)	7LXY		7LXY	
Model resolution (Å)	4.3	4.2	2.9	3.9
FSC threshold	0.5	0.5	0.5	0.5
Model composition				
Nonhydrogen atoms	18,594	558	20,025	759
Protein residues	2,658	75	2,811	96
Glycan residues	42	0	42	0
B factors (Å^2)				
Protein	33	33	31	19
Glycans	36	-	29	-
R.m.s. deviations				
Bond lengths (Å)	0.01	0.01	0.01	0.01
Bond angles (°)	1.0	0.9	1.0	0.8
**Model Validation**				
MolProbity score	0.9	0.5	0.9	0.9
Clashscore	1.3	0.0	1.4	1.9
Rotamer outliers (%)	0.4	0.0	0.5	0.0
Ramachandran plot				
Favored (%)	98	100	98	100
Allowed (%)	2	0	2	0
Outliers (%)	0	0	0	0
EMRinger score	1.4	0.7	4.6	4.1
**Data Availability**				
EMDB	XXXX	XXXX	XXXX	XXXX
PDB	XXXX	XXXX	XXXX	XXXX

bioRxiv preprint doi: https://doi.org/10.1101/2024.10.23.619923; this version posted October 24, 2024. The copyright holder for this preprint (which was not certified by peer review) is the author/funder, who has granted bioRxiv a license to display the preprint in perpetuity. It is made available under aCC-BY 4.0 International license.

Table S4. Cryo-EM data collection, refinement and validation statistics for CMV, HHV6B, HSV1, and VZV gB constructs.

	**CMV gB-deliDoP I5350A2a**		**HHV6B gB-deliDoP I5350A2a**		**HSV1 gB-deliD**		**VZV gB-deliD**
**Data collection and processing**							
Magnification (nominal)	130,000		130,000		45,000		130,000
Voltage (kV)	300		300		200		300
Electron exposure (e^{-}/Å^2)	63		63		47		63
Defocus range (μm)	0.3-2.0		0.3-2.0		0.3-2.0		0.3-2.0
Pixel size (Å)	0.829		0.829		0.89		0.843
Processing Type	Global	Local gB DII-DV	Global	Global	Local (closed)	Local (open)	Global
Symmetry imposed	C1	C3	C3	C3	C3	C3	C3
Initial particle images (no.)	51,511	33,533	85,490	67,753	138,213	138,213	251,305
Final particle images (no.)	33,533	33,533	67,753	67,753	43,635	40,021	48,271
Map resolution (Å)	6.2	5.8	4.3	4.6	5.8	6.3	2.9
FSC threshold	0.143	0.143	0.143	0.143	0.143	0.143	0.143
Map sharpening *B* factor (Å^2)	-461	-455	-113	-251	-362	-442	-96
**Model Refinement**							
Initial model used (PDB code)	7KDP		7KDP		6Z9M		7K1S
Model resolution (Å)	7.9*	7.2*	4.5*	6.7*	8.4*	8.4*	3.3
FSC threshold	0.5	0.5	0.5	0.5	0.5	0.5	0.5
Model composition							
Nonhydrogen atoms	13,049	5,802	10,055	2,747	8,451	8,538	25,656
Protein residues	2,482	1086	1920	507	1650	1,632	3,342
Glycan residues	33	21	25	12	6	18	18
B factors (Å^2)							
Protein	200	122	300	101	197	196	22
Glycans	233	117	388	100	202	217	25
R.m.s. deviations							
Bond lengths (Å)	0.01	0.01	0.01	0.01	0.01	0.01	0.01
Bond angles (°)	1.02	0.96	1.09	1.02	0.98	1.09	0.95
**Model Validation**							
MolProbity score	0.6	0.8	0.8	0.7	1.3	1.2	0.9
Clashscore	0.4	0.7	1.1	0.7	1.3	1.7	1.6
Rotamer outliers (%)	0.0	0.0	0.0	0.0	2.6	2.4	0.5
Ramachandran plot							
Favored (%)	98	98	98	99	99	99	99
Allowed (%)	2	2	2	1	1	1	1
Outliers (%)	0	0	0	0	0	0	0
EMRinger score	N/A*	N/A*	N/A*	N/A*	N/A*	N/A*	4.6
**Data Availability**							
EMDB	XXXX	XXXX	XXXX	XXXX	XXXX	XXXX	XXXX
PDB	XXXX	XXXX	XXXX	XXXX	XXXX	XXXX	XXXX

*most side-chains not built in model reducing reliability of EMRinger score and model resolution calculation
